# Fabrication of Fe_3_O_4_ core-TiO_2_/mesoSiO_2_ and Fe_3_O_4_ core-mesoSiO_2_/TiO_2_ Double Shell Nanoparticles for Methylene Blue Adsorption: Kinetic, Isotherms and Thermodynamic Characterization

**DOI:** 10.3390/nano13182548

**Published:** 2023-09-12

**Authors:** Ahmed Mohamed El-Toni, Mohamed A. Habila, Mohamed Sheikh, Mohamed El-Mahrouky, Abdulrhman S. Al-Awadi, Joselito P. Labis, Zeid A. ALOthman

**Affiliations:** 1 King Abdullah Institute for Nanotechnology, King Saud University, Riyadh 11451, Saudi Arabia; aamohammad@ksu.edu.sa (A.M.E.-T.); jlabis@ksu.edu.sa (J.P.L.); 2 Nanomaterials and Nanotechnology Department, Central Metallurgical Research and Development Institute (CMRDI), P.O. Box 87 Helwan, Cairo 11421, Egypt; 3 Chemistry Department, College of Science, King Saud University, Riyadh 11451, Saudi Arabiazaothman@ksu.edu.sa (Z.A.A.); 4 Soil Science Department, College of Food and Agriculture Sciences, King Saud University, Riyadh 11451, Saudi Arabia; eng.mas2009@gmail.com; 5 K.A. Care Energy Research and Innovation Center in Riyadh, King Saud University, Riyadh 11451, Saudi Arabia; alawadi@ksu.edu.sa

**Keywords:** magnetic Fe_3_O_4_, coating, wastewater purification, methylene blue, nanotechnology, adsorption-modeling

## Abstract

Herein, Fe_3_O_4_ core-TiO_2_/mesoSiO_2_ and Fe_3_O_4_ core-mesoSiO_2_/TiO_2_ double shell nanoparticles were prepared by first (R1) and second (R2) routes and applied for the removal of methylene blue. The reported adsorption capacities for R1-0.2, R1-0.4 and R2 samples were 128, 118 and 133 mg.g^−1^, respectively, which were obtained after 80 min as equilibrium contact time, and pH of 6 using a methylene blue concentration of 200 ppm. The adsorption of methylene blue using the prepared Fe_3_O_4_ core-meso SiO_2_/TiO_2_ double shell was analyzed by kinetic and isotherms models. In addition, thermodynamic investigations were applied to assess the spontaneous nature of the process. The obtained results confirmed that the pseudo-second order model is well fitted with the adsorption data and the Freundlich-isotherm assumption suggested a multilayer adsorption mechanism. In addition, results of the thermodynamic investigation indicated that ΔG° was in the range of −2.3 to −6.8 kJ/mol for R1-0.2, −2.8 to −6.3 kJ/mol for R1-0.4 and −2.0 to −5.2 kJ/mol for R2. In addition, the ΔH° and ΔS° values were found in the range of 26.4 to 36.19 kJ.mol^−1^ and 94.9 to 126.3 Jmol^−1^ K^−1^, respectively. These results confirm that the surfaces of Fe_3_O_4_ core-mesoSiO_2_/TiO_2_ and Fe_3_O_4_ core-TiO_2_/mesoSiO_2_ double shell exhibit a spontaneous tendency to adsorb methylene blue from the aqueous solutions. The achieved performance of Fe_3_O_4_ core-meso SiO_2_/TiO_2_ and Fe_3_O_4_ core-TiO_2_/meso SiO_2_ double shell as adsorbent for methylene blue removal will encourage future research investigations on the removal of a broad range of contaminants from wastewater.

## 1. Introduction

The problems related to water pollution have become the most global environmental issue during the recent decades [1,2,3]. These problems are owed to excessive industrial activities, which cause continuous production of polluted liquid effluents. For example, there are many types of dyes that are produced in huge amounts annually and applied in the textile fields. Part of these tremendous tones of dyes are contaminated with discharge effluents [4,5]. These dyes are dispersed in various environmental components and are hazardous to humans and animals. Furthermore, these pollutants have resulted in a bad aesthetic view and damage the water ecosystem [4,6,7]. Methylene blue (MB) dye is known as methylthionium chloride and classified as a cationic dye [8]. The common industrial applications of methylene blue are in the painting and paper industries, textiles fabrication, pesticides production and pharmaceutical products [9]. The water discharge from these industrial sections contains huge amounts of methylene blue dye, which spreads in the environment. Exposure to methylene blue causes negative impacts on human health such as vomiting, headache, cyanosis, jaundice, quadriplegia, shock and others [10]. A large amount of dye >7.0 mg kg^−1^ causes mental disturbance, abdominal pain and nausea [11].

Currently, the most important methods for methylene blue removal are adsorption, biodegradation, chemical oxidation, photodegradation and membrane filtration. Among these treatment methods, the adsorption process has shown unique advantages such as low cost, easy performance and high removal efficiency [12,13,14]. The common adsorbent materials are zinc oxide, silica and silica-derived materials, alumina and carbon. The broader materials categorized as adsorbents include fly ash, manganese oxide, nickel oxide and transition metal hydroxide, which poses high potential for pollution remediation by adsorption [1,15]. However, the materials in the nanosize exhibit a high surface area and fast dispersion in the adsorption medium, leading to promised adsorption capacity compared with the traditional materials. The application of these non-magnetic nanomaterials is effective wastewater treatment; this has some limitations, such as the need for high speed centrifuge or nanofiltration to separate the adsorbent from the adsorption medium at the end of the treatment process [16,17]. To overcome this limitation, magmatic materials are introduced as adsorbents, which enable high dispersion, porous structures as well as the possibility to be separated by external magnetic field [18]. Furthermore, magnetic-based nanomaterials can be prepared in a core-shell structure, which allows easy functionalization with organic and/or inorganic species. Core-shell-based nanomaterials open the space for tremendous adsorbent materials with amazing abilities regarding adsorption and separation [19,20,21].

Different roots have been developed to prepare core-shell-based magnetic materials; however, the application of Fe_3_O_4_ nanoparticles as the core is most effective due to their superior magnetic properties [22,23,24]. The shell structure can be prepared by various coatings of silica, carbon, polymer or titania to protect the magnetic core and enable various functionalizations [25,26,27]. Salamat et al. have synthetized the Fe_3_O_4_(np)@TiO_2_ shell structure for water treatment applications by photocatalytic degradation of organic pollutants [28]. Shi et al. prepared a core-shell structure of Fe_3_O_4_@titanate using in situ growth and hydrothermal-assisted etching for application in wastewater treatment [29]. Zheng et al. prepared Fe_3_O_4_@ZIF-8 nanoparticles as the core-shell nanostructure and recommended them for the removal of methylene blue with an adsorption capacity of 20.2 mg.g^−1^ [30]. Saini et al. applied Fe_3_O_4_@Ag/SiO_2_ as a core-shell with excellent adsorption properties for the removal of about 99.6% of methylene blue dye from an aqueous solution of pH 7, and the adsorption mechanism was supported by the Langmuir isotherm assumption, reporting a maximum monolayer adsorption capacity (Q_max_) of 128.5 mg/g [31]. Jaseela et al. prepared inorganic–organic adsorbents including TiO_2_ and PVA for selective adsorption of methylene blue with a removal efficiency of 97.1% of MB. The adsorption kinetic was fitted with a pseudo-second-order-based model [32]. Zhan et al. produced an Fe_3_O_4_-derived organic–inorganic hybrid-based adsorbent with various structured magnets (np) by a solvothermal and chemical-based co-precipitation method, naming the products as S-Fe_3_O_4_ and C-Fe_3_O_4_, respectively. The magnetic materials (S-Fe_3_O_4_ and C-Fe_3_O_4_) were further functionalized by dopamine (DA) and (3-aminopropyl) triethoxysilane (KH550) to finally produce the core-shell Fe_3_O_4_/poly(DA + KH550) adsorbents. The application of these materials for methylene blue removal showed an adsorption capacity higher than 400.00 mg.g^−1^, which was well fitted for the pseudo-second-order kinetic model and Langmuir isotherm model [33]. Schneider et al. fabricated an adsorbent composite from Fe_3_O_4_@SiO_2_@carbon for methylene blue removal [34]. Akbarbandari et al. developed bi-metallic and tri-metallic metal–organic frameworks (MOFs) supported on magnetic activated carbon (MAC), which were synthesized for methylene blue removal. The adsorption process was reported to follow the pseudo-second-order kinetic model and Langmuir isotherm model with a maximum adsorption capacity of 66.51 and 71.43 mg/g for the bi-metallic- and tri-metallic-based magnetic nanocomposites, respectively [35]. All these studies [28,29,30,31,32,33,34,35] reported effective methods for wastewater treatment. However, research is still continuing to investigate the various roots for building magnetic core-shell-based nanocomposites with porous structures and different shell combinations of metal oxides to tune the properties of core-shell materials and improve their performance as adsorbents. The novelty of this work is to investigate the effect of ordering during coating titania or silica nanoparticles onto the Fe_3_O_4_ core in relation to adsorption of methylene blue. Therefore, this work aimed to investigate various roots for fabrication of Fe_3_O_4_ core-meso SiO_2_/TiO_2_ double shells for methylene blue adsorption. The structure of the fabricated Fe_3_O_4_ is designed to achieve coating and protection of the magnetic Fe_3_O_4_ core and to produce a double shell around it to enhance the efficiency for methylene removal by the adsorption process. In addition, we study the kinetic, isotherm and thermodynamic properties for the adsorptive removal of methylene blue.

## 2. Materials and Methods

All applied chemicals were of high-purity analytical grade. Ferric chloride-hexahydrate (FeCl_3_.6H_2_O), sodium acetate, sodium citrate, ammonia solution, TBOT, TEOS and cetyltrimethylammonium bromide were obtained from Sigma-Aldrich (St. Louis, MO, USA).

### 2.1. Synthesis of Fe_3_O_4_ Magnetic Core

A certain weight of FeCl_3_·6H_2_O was dissolved in a certain volume of ethylene glycol; then, calculated amounts of sodium acetate, tri-sodium citrate and polyethylene glycol were added. The mixture was vigorously and continuously stirred to ensure complete mixing; then, it was transferred to an autoclave made of stainless steel, which was lined with Teflon and heated to around 190 °C for a certain time. At the end, after reaching room temperature, the produced Fe_3_O_4_ magnetic core was washed three times with ethanol and then dried at 60 °C in an oven for approximately 6 h [36].

### 2.2. Synthesis of Fe_3_O_4_ core-meso SiO_2_/TiO_2_ Double Shell Nanoparticles

#### 2.2.1. Coating with Mesoporous Silica

Fe_3_O_4_ core magnetic nanoparticles were coated with mesoporous silica shell according to the following procedure. Fe_3_O_4_ was dispersed in H_2_O/ethanol mixture ultrasonically; then, an exact volume of NH_4_OH was added. Thereafter, a specific weight of the cationic surfactant (cetyltrimethylammonium bromide) was added, followed by the addition of TEOS [37].

#### 2.2.2. Titania Coating

To make a layer of titania onto the magnetic nanocores, the procedure described in the work of Jianping et al. [38] was applied. In detail, the silica-coated magnetic nanocores were dispersed in ethanol and mixed with ammonia solution under ultrasonic stirring. TBOT was then added slowly. The reaction was allowed to continue for 24 h under mechanical stirring. Thereafter, the formed Fe_3_O_4_ core-SiO_2_/TiO_2_ double shell nanoparticles were separated from the mother solution, washed several times with de-ionized water and then with ethanol, dried and finally calcined at 500 °C for 2 h to form Fe_3_O_4_ core-meso SiO_2_/TiO_2_ double shell nanoparticles.

### 2.3. Synthesis of Multifunctional Fe_3_O_4_ core-TiO_2_/meso SiO_2_ Double Shell Nanoparticles

For fabrication of Fe_3_O_4_ core-TiO_2_/meso SiO_2_ double shell nanoparticles, the titania layer was coated onto the magnetic Fe_3_O_4_ core nanoparticle surface; then, a silica coating was made as a second layer and the samples were finally calcined. In detail, the magnetic nanocores were dispersed in ethanol and mixed with ammonia solution under ultrasonic stirring. Then, TBOT was added slowly (0.2 and 0.4 mL). The reaction was allowed to continue under mechanical stirring for 24 h. Thereafter, the produced Fe_3_O_4_ core-TiO_2_ shell was separated from the mother solution, washed several times with de-ionized water and then with ethanol, dried and finally calcined at 500 °C for 2 h under air condition. The final step for coating meso SiO_2_ onto Fe_3_O_4_ core-TiO_2_ was applied according to the following procedure: Fe_3_O_4_@TiO_2_ as a core was dispersed in H_2_O ultrasonically and the exact volume from NH_4_OH was added. Thereafter, the cationic surfactant (cetyltrimethylammonium bromide) solution was added, followed by the addition of TEOS [38]. The reaction was allowed to continue under mechanical stirring for 6 h. The formed Fe_3_O_4_ core-TiO_2_/mesoSiO_2_ double shell nanoparticles were separated from the mother solution and washed with ethanol and water. Finally, the samples were calcined at 500 °C for 2 h to form Fe_3_O_4_ core-TiO_2_/meso SiO_2_ double shell nanoparticles.

### 2.4. Adsorptive Removal Study for Methylene Blue

To investigate the fabricated Fe_3_O_4_ core-meso SiO_2_/TiO_2_ double shell for methylene blue uptake, the batch process was applied. A certain weight of fabricated Fe_3_O_4_ core-meso SiO_2_/TiO_2_ double shell was taken in a 50 mL tube and mixed with 25 mL of the 200 ppm methylene blue dye solution. The mixture was shaken for 80 min; then, the phases were separated by external magnetic field. The concentration of the methylene blue dye was measured by UV–Visible. Blank samples without fabricated Fe_3_O_4_ core-meso SiO_2_/TiO_2_ double shell were investigated in all experiments. The adsorption capacity (qe) was calculated from Equation (1):

qe = (C_0_ − Ce) × V/M, (1)

where C_0_ is the primary concentration of methylene blue solution, Ce is the final methylene blue concentration, V represents the volume of the adsorption solution and M is the adsorbent mass (g) (Fe_3_O_4_ core-meso SiO_2_/TiO_2_ double shell).

The procedures for the adsorption of methylene blue onto Fe_3_O_4_ core-meso SiO_2_/TiO_2_ double shell were repeated to investigate the most import factors such as pH, contact time, methylene blue dye concentration and temperature, which significantly affect the uptake of methylene blue onto fabricated Fe_3_O_4_ core-meso SiO_2_/TiO_2_ double shell. For reusability in investigations, the applied Fe_3_O_4_ core-meso SiO_2_/TiO_2_ double shells were washed three times each with 3 mL ethanol and reused directly.

## 3. Results and Discussion

### 3.1. Characterization of Fe_3_O_4_ core-Double Shell

**Figure 1:**
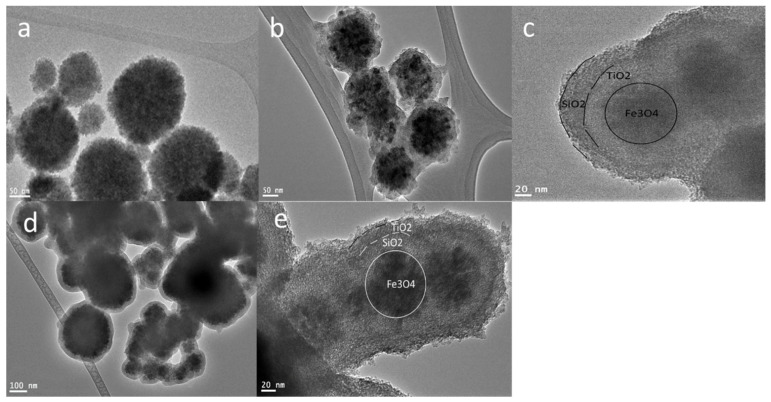
TEM images of (**a**) Fe_3_O_4_ nanocores prepared by solvothermal method, (**b**) titania-coated Fe_3_O_4_ nanocores and (**c**) double shell Fe_3_O_4_@TiO_2_@m-SiO_2_ by first route (R1), and (**d**) mesoporous silica-coated Fe_3_O_4_ nanocores and (**e**) double shell Fe_3_O_4_@m-SiO_2_@TiO_2_ by second route (R2).

Multiple procedures were applied to prepare Fe_3_O_4_ core-meso SiO_2_/TiO_2_ double shells with alternative sequences of titania and silica. In the first stage, the homogenous spherical magnetic noncore (Fe_3_O_4_) was obtained via a solvothermal process, which resulted in separated particles with an average size between 50 nm and 100 nm (Figure 1a). The obtained magnetic nanocore was applied to fabricate the Fe_3_O_4_ core-mesoSiO_2_/TiO_2_ double shell or Fe_3_O_4_ core-TiO_2_/mesoSiO_2_ double shell.

To fabricate Fe_3_O_4_@TiO_2_@m-SiO_2_, we used Fe_3_O_4_ nanoparticles with the first route. The nanoparticles were firstly coated with TiO_2_ shell and secondly with mesoporous silica shell; finally, the calcination process was conducted to crystalize the TiO_2_ shell and to remove surfactants from the silica shell to transform it into a mesoporous one. TiO_2_ coating onto Fe_3_O_4_ nanoparticles was conducted using the Stöber-modified approach, where citrate-modified Fe_3_O_4_ nanoparticles were dispersed in ethanol solution, ammonium hydroxide and titanium butoxide were added to the above mixture and then the coating process was conducted at 45 °C for 20 h. The mesoporous silica step was conducted on TiO_2_-coated Fe_3_O_4_ using the Stöber approach by adding a cationic surfactant (Cetyl trimethylammonium bromide (CTAB)). Finally, the calcination process was conducted to ensure the crystallization of the TiO_2_ shell and to remove CTAB to obtain a mesoporous silica shell. TEM observation (Figure 1b) showed the formation of a ~25 nm TiO_2_ layer around Fe_3_O_4_ nanocores. The TEM image (Figure 1c) revealed that the Fe_3_O_4_@TiO_2_@m-SiO_2_ structure was formed with a shell thickness of 20 nm. In route R1, two different samples Fe_3_O_4_@TiO_2_@m-SiO_2_ -0.2(R1-0.2) and Fe_3_O_4_@TiO_2_@m-SiO_2_ -0.4(R1-0.4) were synthesized by adding 0.2 and 0.4 mL of TEOS, respectively. The thickness of the shell layer was 20 and 45 nm for the R1-0.2 and R2-0.4 samples, respectively.

To fabricate the Fe_3_O_4_ core-mesoSiO_2_/TiO_2_ double shell using the second route (sample R2), the magnetic nanocores were subjected first to mesoporous silica coating and then to TiO_2_ coating for the formation of the second shell. To achieve uniform formation of Fe_3_O_4_@mesoSiO_2_, the Stöber method in the presence of cationic surfactant was applied to form a mesoporous silica layer around the magnetic nanocores followed by titania coating, as shown in Figure 1d. The mesoporous silica layer is about 20 nm (Figure 1d). The second step to coat TiO_2_ on the fabricated Fe_3_O_4_ core-meso SiO_2_ was achieved by hydrolysis of titanium butoxide, which successfully formed a uniform 20 nm layer of TiO_2_ (Figure 1e) to finally form the Fe_3_O_4_ core-meso SiO_2_/TiO_2_ double shell (Figure 1). Finally, calcining of the Fe_3_O_4_ core-meso SiO_2_/TiO_2_ double shell sample at 550 °C was performed to crystallize the TiO_2_ layer and to remove the surfactant in one single step.

**Figure 2:**
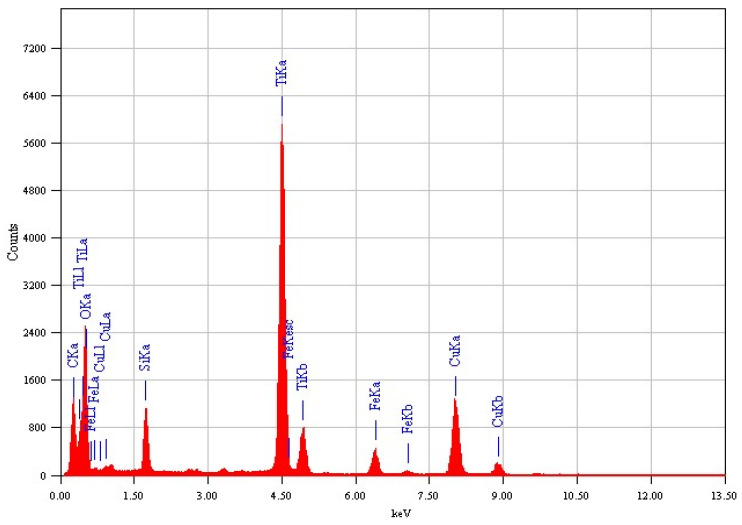
Elemental EDX analysis of Fe_3_O_4_@TiO_2_@m-SiO_2_ by first route (R1).

**Figure 3:**
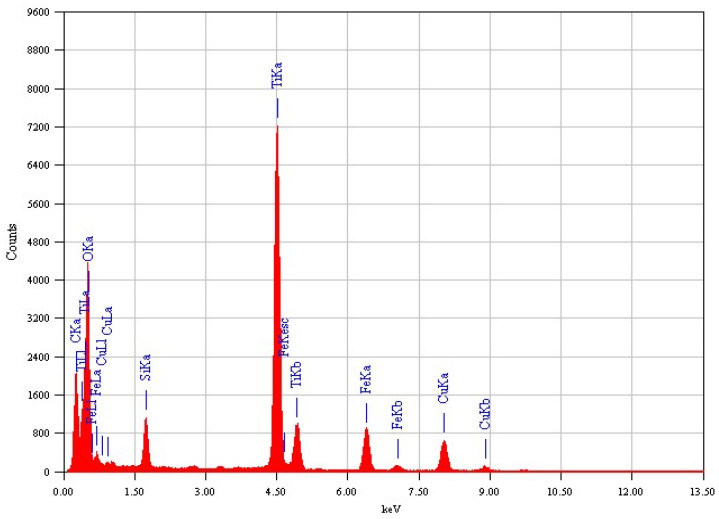
Elemental EDX analysis of Fe_3_O_4_@m-SiO_2_@TiO_2_ by second route (R2).

The EDX analysis for both samples, Fe_3_O_4_@TiO_2_@m-SiO_2_ and Fe_3_O_4_@m-SiO_2_@TiO_2_, are shown in Figure 2 and Figure 3, respectively. The detected elements indicate the formation of the desired core-double shell structure.

**Figure 4:**
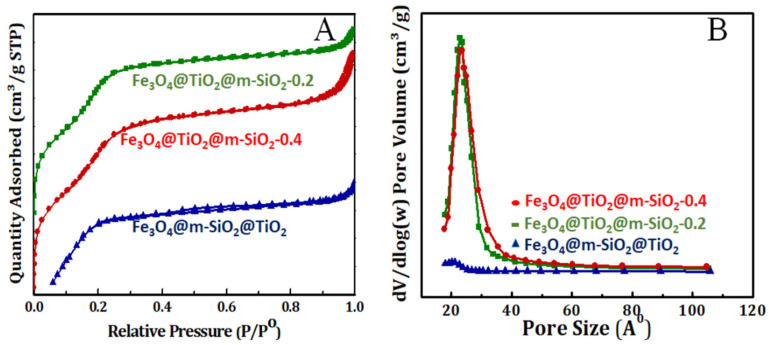
(**A**) N_2_ sorption isotherm and (**B**) pore size distribution of calcined Fe_3_O4@TiO_2_@m-SiO_2_ at TEOS amounts of 0.2 and 0.4 mL prepared by route 1 (R1) and calcined Fe_3_O_4_@m-SiO_2_@TiO_2_ prepared by route 2 (R2).

N_2_ adsorption–desorption isotherms were conducted for core-double shell nanoparticles prepared by route 1 (at 0.2 and 0.4 mL TEOS) and route 2 at 77 K and presented in Figure 4. The prepared core-double shell derived nanoparticles by route 1 and route 2 exhibited a porous structure with type IV isotherm. It is clear that nanoparticles prepared by route 1, with the silica shell outer layer, had higher surface areas as well as larger pore volumes (Table 1 and Figure 4A) when compared with nanoparticles prepared by route 2, where the TiO_2_ shell is the outer layer. Moreover, these results can be explained based on the porous character of the silica shell compared with the crystalline dense character of the TiO_2_ one. However, changing the silica content caused a slight increment in the surface area and pore volume of the formed sample. Moreover, the pore size was much bigger in the case of core-double shell nanoparticles prepared by route 1 than in samples by route 2 (Table 1 and Figure 4B). In both samples, Fe_3_O_4_@TiO_2_@m-SiO_2_ by first route (R1) and Fe_3_O_4_@m-SiO_2_@TiO_2_ by second route (R2), the heat-treatment process was conducted with an aim to convert amorphous TiO_2_ to a crystalline one and to remove surfactant molecules from the silica shell to impart it with a mesoporous character. The heat-treatment process is our confirmation of the crystalline character of the TiO_2_ shell. Second, the amorphous TiO_2_ shell was built on silica layer by controlled hydrolysis of titanium (IV) butoxide (TBOT). After formation of the amorphous TiO_2_ shell on the silica middle layer, the heat-treatment process allows us to convert it to a crystalline dense character.

**Figure 5:**
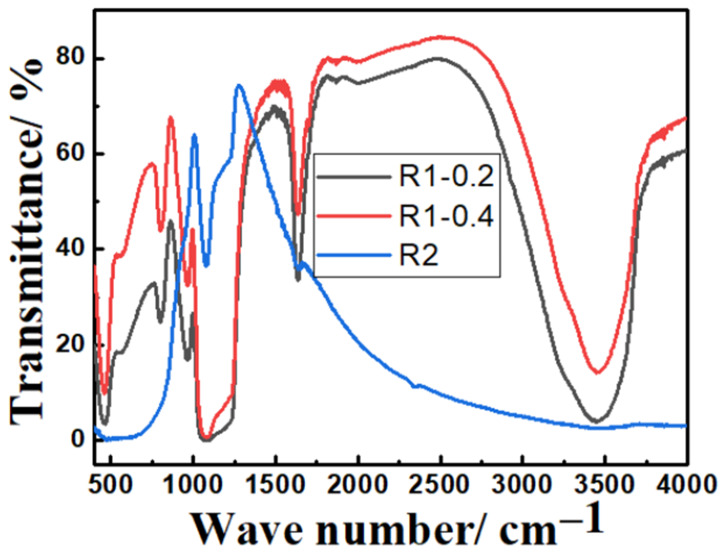
FTIR spectra of calcined Fe_3_O_4_@TiO_2_@m-SiO_2_ at TEOS amounts of 0.2 and 0.4 mL prepared by route 1 (R1) and calcined Fe_3_O_4_@m-SiO_2_@TiO_2_ prepared by route 2 (R2).

FTIR measurements of core-double shell nanoparticles prepared by route1 (at 0.2 and 0.4 mL TEOS) and route 2 are shown in Figure 5. The Si-O peak can be seen at 1050–1250 cm^−1^. The Fe–O–Si peak that refers to chemical binding between Fe_3_O_4_ and silica cannot be seen in the FTIR spectrum because it appears at around 584 cm^−1^ and, therefore, overlaps with the Fe–O vibration of magnetite nanoparticles. The peaks at 1632 cm^−1^ and 3425 cm^−1^ correspond to the vibration of hydroxyl groups (-OH) on the surface of Fe_3_O_4_ nanoparticles. The peak at 970 cm^−1^ can be attributed to the Ti–O–Si bond while the shoulder at 1400 cm^−1^ can be due to the Ti–O–Ti vibration.

### 3.2. Adsorptive Remediation Investigation

**Figure 6:**
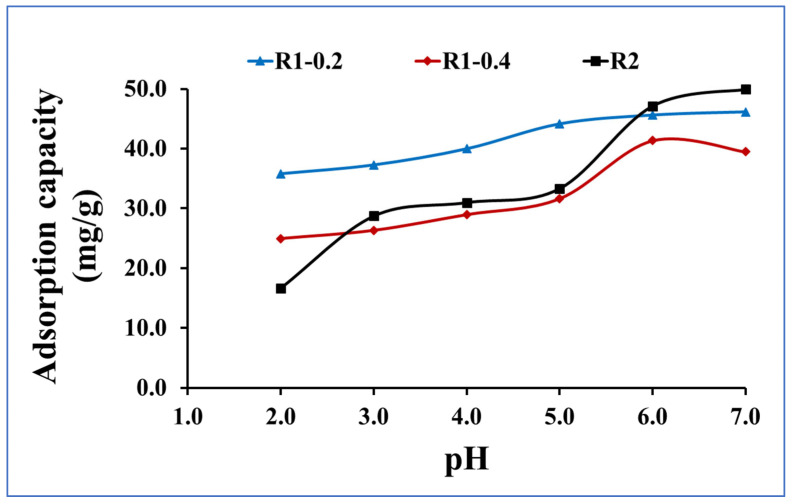
pH investigation for methylene blue adsorption onto R1–0.2, R1-0.4 and R2 (methylene blue concentration 100 mg L^−1^, adsorbent dose 0.015 g and contact time 120 min).

Methylene blue is extensively used in the industrial section for dying and painting, resulting in huge amounts of colored discharge and producing many negative environmental impacts [39,40,41]. Herein, three adsorbent materials including the core-double shell structures from Fe_3_O_4_ core-TiO_2_/mesoSiO_2_ (R1–0.2), Fe_3_O_4_ core-TiO_2_/mesoSiO_2_ (R1-0.4) and Fe_3_O_4_ core-mesoSiO_2_/TiO_2_ (R2) double shell nanoparticles were applied for methylene blue dyes by adsorptive removal. The effect of the pH of the medium was investigated by varying the pH of the methylene blue sample solution from 2 to 7 (Figure 6). In the strong acidic medium, the adsorption capacities for methylene blue removal using R1–0.2, R1-0.4 and R2 had the lowest values; then, they increased with the increasing pH, reaching its maximum value between pH 6 and 7. The lower adsorption capacity at strong pH medium may be owed to the protonation of the adsorbent surfaces [42,43]. The adsorption capacity of methylene blue onto the three tested adsorbents was in the order of R2 > R1–0.2 > R1-0.4. This indicates that the titania as outer shell was more effective for adsorptive removal of methylene blue. This may be attributed to the vacant d orbital in the TiO2 structure, which can correlate and interact with the lone pairs of electrons on the methylene blue structure leading to stronger attractive forces than in the case of silica as the outer shell. The blank experiments that operated in the absence of Fe_3_O_4_ core-TiO_2_/mesoSiO_2_ or Fe_3_O_4_ core-mesoSiO_2_/TiO_2_ double shell nanoparticles showed zero efficiency for the adsorption of methylene blue, suggesting that no other mechanisms such as precipitation or coagulation are involved during the developed wastewater treatments. This proves the effective performance of Fe_3_O_4_ core-TiO_2_/mesoSiO_2_ or Fe_3_O_4_ core-mesoSiO_2_/TiO_2_ double shell nanoparticles as adsorbents to remove methylene blue dye from wastewater.

**Figure 7:**
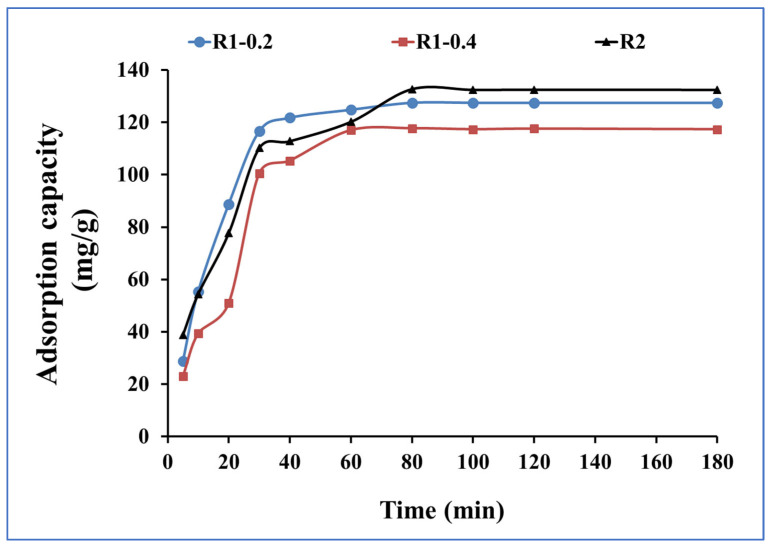
Time investigation for methylene blue adsorption onto R1–0.2, R1-0.4 and R2 (methylene blue concentration 200 mg L^−1^, adsorbent dose 0.015 g and pH 7).

The effect of contact time on the de-colorization of dyes from aqueous solution by adsorption was investigated to assess the rate and efficiency of the process [44,45]. The effect of time is studied from 5 min to 180 min and the adsorption capacities for R1–0.2, R1-0.4 and R2 for methylene blue uptake are presented in Figure 7. The adsorption capacities after 5 min were 29, 23 and 39 mg.g^1^ for R1–0.2, R1-0.4 and R2, respectively; then, they increased until reaching equilibrium at 80 min, recording adsorption capacities of 128, 118 and 133 mg.g^1^. When increasing time from 80 min to 180 min, no noticeable improvements in the adsorption capacities were detected due to the occurrence of the steady state.

**Figure 8:**
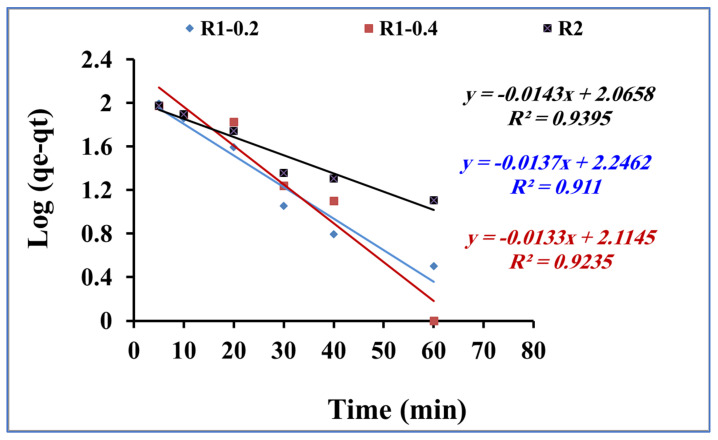
Pseudo-first-order kinetic model for methylene blue adsorption using R1-0.2, R1-0.4 and R2 (methylene blue concentration 200 mg L^−1^, adsorbent dose 0.015 g and pH 7).

**Figure 9:**
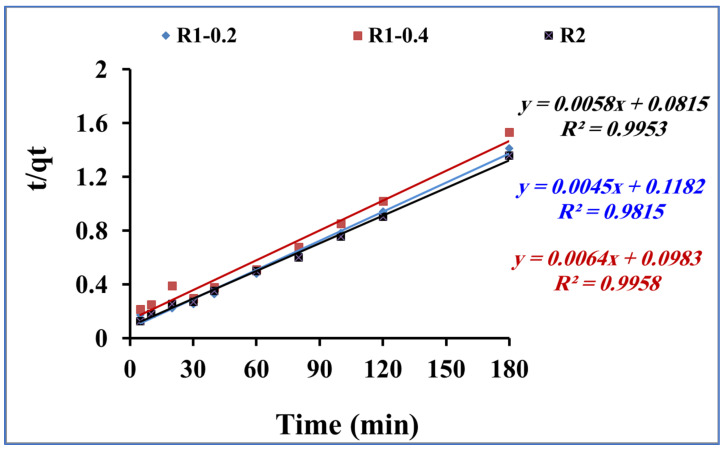
Pseudo-second-order kinetic model for methylene blue adsorption using R1–0.2, R1-0.4 and R2 (methylene blue concentration 200 mg L^−1^, adsorbent dose 0.015 g and pH 7).

The rate of the mass transfer of methylene blue during the adsorption process onto R1–0.2, R1-0.4 and R2 was studied by applying the kinetic models of pseudo first order and pseudo second order [46,47], as presented in Figure 8 and Figure 9, respectively. From the data correlation, the pseudo-second-order kinetic model was found to be more comfortable for describing the rate of the adsorption process. The pseudo-first-order equation of Lagergren is generally expressed in the integrated form of Equation (2):

log(qe − qt) = log qe − k1t/2.303(2)

By plotting log (qe − qt) versus time t (Figure 8), the pseudo-first-order rate constant k1 is calculated and reported in Table 2. In addition, the pseudo-second-order kinetic rate equation is expressed in the integrated form of Equation (3):

t/qt = 1/Kqe2 + 1/qe.t(3)

where t is the time (min), and qe (mg/g) and qe2 (mg/g) are the quantity of methylene blue adsorbed at equilibrium onto fabricated R1–0.2, R1-0.4 and R2 samples at pH 6 and 25 °C. Figure 9 presents the plotting of t/qt versus t. The qe and k parameters are calculated using the slope and intercept, respectively, according to the second-order kinetic model (Equation (3)).

Table 2 shows the calculated parameter values and the linear regression correlation coefficient values. The pseudo-second-order kinetic rate equation model fitting was much better than the pseudo-first-order one. The obtained results confirm the assumption related to the second-order kinetic model, including the fast adsorption process. In addition, the adsorption dynamic is dependent on the migration of the methylene blue molecules to the surface of the fabricated Fe_3_O_4_ core-meso SiO_2_/TiO_2_ double shell adsorbent and, finally, migration of the methylene blue molecules to the entire pores of the fabricated Fe_3_O_4_ core-meso SiO_2_/TiO_2_ double shell adsorbent [48,49].

### 3.3. Isotherms Study

**Figure 10:**
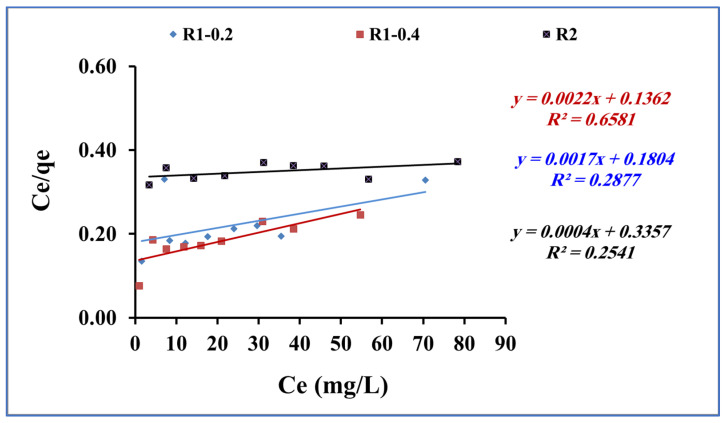
Langmuir for methylene blue adsorption using R1-0.2, R1-0.4 and R2 (methylene blue concentration 200 mg L^−1^, adsorbent dose 0.015 g, pH 7 and contact time of 80 min).

The investigation of the effect of the concentration of methylene blue at constant temperatures (isotherms) on the adsorption capacity using fabricated R1-0.2, R1-0.4 and R2 samples is applied to assess the distribution of methylene blue as the adsorbate within the liquid sample solution and the solid fabricated Fe_3_O_4_ core-meso SiO_2_/TiO_2_ double shell adsorbent at equilibrium [50,51,52,53]. The Langmuir Equation (4) was used to model the adsorption data for methylene blue uptake onto the fabricated Fe_3_O_4_ core-meso SiO_2_/TiO_2_ double shell adsorbent (R1-0.2, R1-0.4 and R2 samples) [54]:

Ce/Qe = 1/(qmax. b) + Ce/qmax),(4)

where Ce is the concentration of methylene blue (mg/L) at equilibrium, Qe is the quantity of methylene blue adsorbed (mg/g), and qmax and b are Langmuir model constants (Figure 10). The correlation coefficients, R^2^, for adsorption data for methylene blue adsorption onto fabricated R1-0.2, R1-0.4 and R2 samples were low (Table 3), indicating that the adsorption data were not fitted by the Langmuir isotherm.

**Figure 11:**
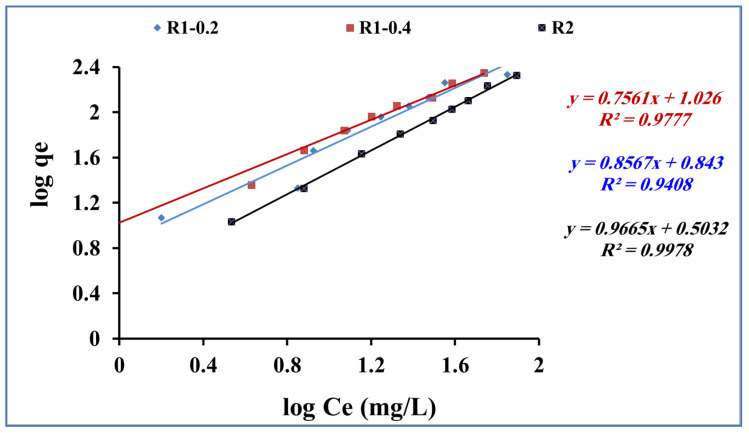
Freundlich isotherm for methylene blue adsorption onto using R1-0.2, R1-0.4 and R2 (methylene blue concentration 200 mg L^−1^, adsorbent dose 0.015 g, pH 7 and contact time of 80 min).

The Freundlich model assumes that the adsorption process occurs as multiple layers of adsorbate molecules (methylene blue) onto the surface of the adsorbent (Fe_3_O_4_ core-meso SiO_2_/TiO_2_ double shell). The obtained adsorption data for methylene blue adsorption onto fabricated R1-0.2, R1-0.4 and R2 samples were subjected to Freundlich equation [55] (Equation (5)):

Log qe = log K_f_ + 1/n log Ce, (5)

where Ce is the concentration of methylene blue (mg/L) at equilibrium and Qe is the quantity of methylene blue adsorbed (mg/g). K_F_ (mg/g) is the Freundlich constant for the adsorbent capacity and n is related to the favorable nature of the adsorption process. Figure 11 shows the plotting of log qe and log Ce. From Freundlich equation and Figure 11, the slope and intercept indicate 1/n and log K_F_, respectively.

The adsorption of methylene blue using Fe_3_O_4_ core-meso SiO_2_/TiO_2_ double shell (R1-0.2, R1-0.4 and R2 samples) showed agreement with the Freundlich model (R_2_ > 0.9) for the tested range of concentrations used in this study (Table 3), suggesting a multilayer adsorption process.

### 3.4. Thermodynamic Studies

The adsorption process is strongly influenced by the temperature of the adsorption medium [56,57,58]. The temperature effect has been studied to evaluate the nature of the adsorption process of methylene blue onto the fabricated Fe_3_O_4_ core-meso SiO_2_/TiO_2_ double shell. The thermodynamic parameters, including the Gibbs free energy (ΔG°), enthalpy (ΔH°) and entropy (ΔS°) of the adsorption process of methylene blue, onto the fabricated R1-0.2, R1-0.4 and R2 samples are evaluated by Equations (6) and (7):

logKd = ΔS°/2.303R − ΔH°/2.303RT(6)

**Figure 12:**
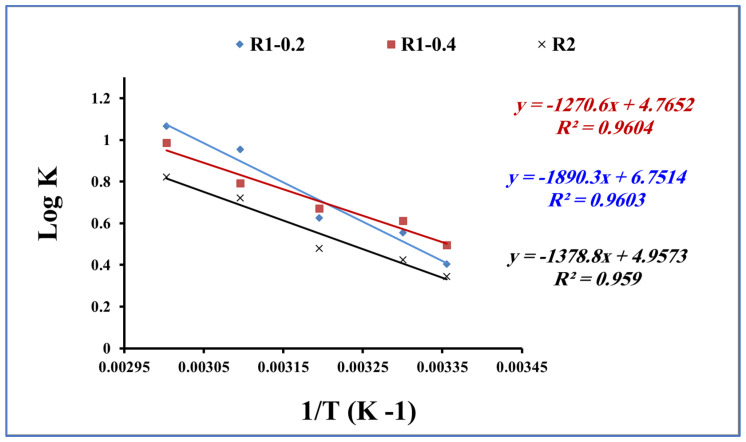
Thermodynamic parameters for methylene blue adsorption using R1-0.2, R1-0.4 and R2 (methylene blue concentration 200 mg L^−1^, adsorbent dose 0.015 g, pH 7 and contact time of 80 min).

ΔG° = −RT lnKd,(7)

where K_d_ refers to the equilibrium partition constant, which is calculated as the ratio between the sorption capacity of Fe_3_O_4_ core-meso SiO_2_/TiO_2_ double shell (qe) and methylene blue equilibrium concentration (Ce), R represents the gas-constant (8.314 J/mol K) and T is the temperature in Kelvin (K). Equation (6) and the plot of log K_d_ and 1/T (Figure 12) enable the calculation of ΔH° and ΔS° values.

Gibbs free energy (ΔG°), enthalpy (ΔH°) and entropy (ΔS°) are presented in Table 4. ΔG° was obtained as negative values in the range of −2.3 to −6.8 kJ/mol for R1-0.2, −2.8 to −6.3 kJ/mol for R1-0.4 and −2.0 to −5.2 kJ/mol for R2. In addition, the ΔH° and ΔS° values were found in the range of 26.4 to 36.19 kJ.mol^−1^ and 94.9 to 126.3 Jmol^−1^ K^−1^, respectively. The calculated thermodynamic parameters indicate that the adsorption process of methylene blue onto fabricated R1-0.2, R1-0.4 and R2 samples is spontaneous and physical in nature. Furthermore, the adsorption process of methylene blue increases the degree of freedom during the adsorption interaction process [59,60].

The reported adsorption capacity for the removal of methylene blue using the fabricated Fe_3_O_4_ core-TiO_2_/mesoSiO_2_ and Fe_3_O_4_ core-mesoSiO_2_/TiO_2_ double shell nanoparticles is compared with other studies from the literature [30,31,35,61,62,63] (Table 5). The obtained adsorption capacity achieved in this work is higher than that reported by Zheng et al. onto Fe_3_O_4_@ZIF-8 (20.2 mg.g^−1^) [30], Saini et al. using Fe_3_O_4_@Ag/SiO_2_ (128.5 mg/g) [31], Akbarbandari et al. (66.51 and 71.43 mg.g^−1^ onto bi-metallic and tri-metallic metal–organic frameworks, respectively) [35], Bouyahia et al. (7.84 mg.g^−1^) onto sawdust [61], Kazemi and Sobhani (54.05 mg.g^−1^) onto CuMn_2_O_4_/chitosan micro/nanocomposite [62] and Taweekarn et al. (34.84 mg.g^−1^) onto Monolithic starch cryogel [63]. The obtained results in this work are lower than those reported by Zhan et al. (higher than 400.00 mg.g^−1^) onto Fe_3_O_4_-derived organic/inorganic (S-Fe_3_O_4_ and C-Fe_3_O_4_) [33]. The variation in adsorption efficiencies during wastewater treatments is highly influenced by the nature of the adsorbent materials, porosity and nature of adsorbate [64,65].

**Figure 13:**
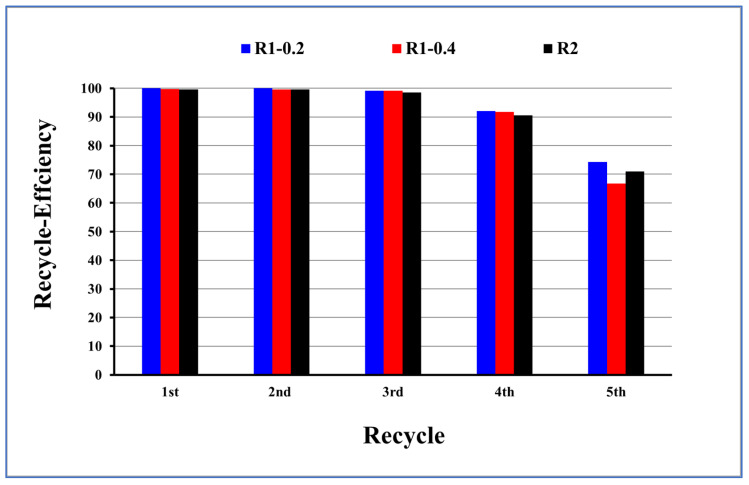
The efficiency of removal of methylene blue using R1-0.2, R1-0.4 and R2 for recycling investigations.

The fabricated Fe_3_O_4_ core-TiO_2_/mesoSiO_2_ and Fe_3_O_4_ core-mesoSiO_2_/TiO_2_ double shell nanoparticles were subjected to reactivation and recycling after adsorption of methylene blue (Figure 13). The efficiency during recycling was calculated as percentage from the first usage. Results presented in Figure 13 indicate that the efficiency for the removal of methylene blue was still above 90% for four usages for both adsorbents; then, the efficiency decreased to below 75% in the fifth usage.

## 4. Conclusions

Multistep fabrication processes were investigated in this study. Fe_3_O_4_ core-TiO_2_/mesoSiO_2_ and Fe_3_O_4_ core-mesoSiO_2_/TiO_2_ double shell nanoparticles were prepared by first (R1) and second (R2) routes as magnetic materials for adsorption of methylene blue. TEM examination showed the successful formation of a magnetic core-double shell structure including silica layer and titania layer of 20 nm thickness. The prepared magnetic core-double shell nanoparticles exhibit surface areas of 1133, 1207 and 52.27 m^2^/g for R1-0.2, R1-0.4 and R2 samples, respectively. The removal of methylene blue was operated at pH 6 with a contact time of 80 min to reach the steady state with an adsorption capacity of 128, 118 and 133 mg.g^−1^ for R1-0.2, R1-0.4 and R2, respectively. Upon applying the kinetic models, the pseudo-second-order kinetic model was well fitted with the adsorption data for the removal of methylene blue onto fabricated Fe_3_O_4_ core-TiO_2_/mesoSiO_2_ and Fe_3_O_4_ core-mesoSiO_2_/TiO_2_ double shell nanoparticles (R1-0.2, R1-0.4 and R2 samples). The Feundlish isotherm showed good correlation with the adsorption data, suggesting a multilayer adsorption. The thermodynamic parameters confirm that the adsorption process of methylene blue onto the fabricated magnetic core-double shell structure is spontaneous and physical in nature.

## Figures and Tables

**Table 1:** Textural properties for magnetic core-double shell prepared by routes 1 and 2.

Sample	BET S. A. m^2^/g	Pore Volume cm^3^/g	Pore Size A°
Fe_3_O_4_@TiO_2_@m-SiO_2_-0.2 (R1-0.2)	1133.28	0.74	25.79
Fe_3_O_4_@TiO_2_@m-SiO_2_ (R1-0.4)	1207.49	0.88	29.86
Fe_3_O_4_@m-SiO_2_@TiO_2_ (R2)	52.27	0.03	24.02

**Table 2:** Kinetic constant parameters obtained for methylene blue adsorption using R1–0.2, R1-0.4 and R2 (methylene blue concentration 200 mg L^−1^, adsorbent dose 0.015 g and pH 7).

		Pseudo-First-Order			Pseudo-Second-Order		
	q_e_,exp (mg/g)	K_1_ (min^−1^)	q_e_,cal (mg/g)	R^2^	k_2_ (g/mg.min)	q_e_,cal (mg/g)	R^2^
R1-0.2	128	0.031	176.27	0.91	1.71 × 10^−4^	222.22	0.98
R1-0.4	118	0.03	130.16	0.93	4.16 × 10^−4^	156.25	0.99
R2	133	0.032	116.35	0.92	4.12 × 10^−4^	172.41	0.99

**Table 3:** Langmuir and Freundlich parameters for methylene blue adsorption using R1-0.2, R1-0.4 and R2 (methylene blue concentration 200 mg L^−1^, adsorbent dose 0.015 g, pH 7 and contact time of 80 min).

	Langmuir Constants				Freundlich Constants		
	K_L_	b	Q_max._	R^2^	K_F_	n	R^2^
R1-0.2	5.54	9.4 × 10^−3^	588.2	0.28	6.96	1.16	0.94
R1-0.4	7.34	0.016	454.5	0.65	10.61	1.32	0.97
R2	2.97	1.19 × 10^−3^	2500	0.25	3.18	1.03	0.99

**Table 4:** Thermodynamic parameters for methylene blue adsorption onto R1-0.2, R1-0.4 and R2.

	Temperature T(K)	Thermodynamic Parameters		
		ΔG° (kJ/mol)	ΔS° (J/mol/K)	ΔH° (kJ/mol)
R1-0.2	273	−2.3	129.3	36.19
	278	−3.2		
	288	−3.7		
	298	−5.9		
	308	−6.8		
R1-0.4	273	−2.8	91.2	24.33
	278	−3.5		
	288	−4.0		
	298	−4.9		
	308	−6.3		
R2	273	−2.0	94.9	26.40
	278	−2.5		
	288	−2.9		
	298	−4.5		
	308	−5.2		

**Table 5:** Comparison of removal of methylene blue between achieved performance in this work and other studies from the literature.

Adsorbent Materials	Adsorption Capacity and/or Efficiency%	References
Fe_3_O_4_@ZIF-8 as core–shell nanostructure	20.2 mg.g^−1^	[30]
Fe_3_O_4_@Ag/SiO_2_	128.5 mg.g^−1^	[31]
S-Fe_3_O_4_ and C-Fe_3_O_4_	>400.00 mg.g^−1^	[33]
Bi-metallic-based magnetic nanocomposites	66.51 mg.g^−1^	[35]
Tri-metallic-based magnetic nanocomposites	71.43 mg.g^−1^	[35]
Sawdust	7.84 mg.g^−1^	[61]
CuMn_2_O_4_/chitosan micro/nanocomposite	54.05 mg.g^−1^	[62]
Monolithic starch cryogel	34.84 mg.g^−1^	[63]
Fe_3_O_4_ core-TiO_2_/mesoSiO_2_ -0.2	128 mg.g^−1^	This work
Fe_3_O_4_ core-TiO_2_/mesoSiO_2_ -0.4	118 mg.g^−1^	This work
Fe_3_O_4_ core-mesoSiO_2_/TiO_2_	133 mg.g^−1^	This work

## Data Availability

Data and/or Samples of the compounds are available from the authors.
